# Chasing among older-aged gamblers: the role of mentalizing and psychological distress

**DOI:** 10.3389/fpsyt.2024.1418339

**Published:** 2024-11-13

**Authors:** Maria Ciccarelli, Barbara Pizzini, Marina Cosenza, Francesca D’Olimpio, Mark D. Griffiths, Giovanna Nigro

**Affiliations:** ^1^ Department of Psychology, University of Campania Luigi Vanvitelli, Caserta, Italy; ^2^ Law Department, Giustino Fortunato University, Benevento, Italy; ^3^ Psychology Department, Nottingham Trent University, Nottingham, United Kingdom

**Keywords:** gambling, problem gambling, older-aged gambling, chasing, psychological distress, mentalizing, depression, hypermentalizing

## Abstract

**Background:**

Despite the increasing proportion of older-aged individuals suffering from problematic gambling, research on gambling among this specific age cohort is still in its infancy. Chasing is a pathognomonic feature of disordered gambling and is considered one of the key risk factors in the transition from recreational to disordered gambling. Despite the increased research on chasing over the past decade, no previous study has ever examined the psychological determinants of chasing behavior among old-aged gamblers. Given the importance of chasing in facilitating and maintaining problem gambling, and the paucity of research examining gambling among older individuals, the present study is the first to empirically investigate the joint role of chasing behavior, negative affectivity, and mentalizing among older-aged gamblers.

**Methods:**

The sample comprised 116 older-aged gamblers who were administered the South Oaks Gambling Screen (SOGS), the Depression Anxiety Stress Scale (DASS-21) and the Reflective Functioning Questionnaire (RFQ-8) to assess gambling severity, psychological distress, and mentalizing, respectively. Participants also performed the ChasIT, a computerized task that assesses chasing behavior, in which participants were randomly assigned to three different experimental conditions: loss, control, and win.

**Results:**

No effect of the experimental conditions of ChasIT on chasing behavior was observed. Regression analyses indicated that heightened levels of gambling severity and lower levels of certainty about mental states (i.e., hypermentalizing) predicted both the decision to chase and chasing frequency. Along with problem gambling and hypermentalizing, chasing frequency was also predicted by high levels of depression.

**Conclusions:**

The present study demonstrated the association between disordered gambling, depression, and hypermentalizing in chasing behavior among older-aged gamblers. The findings make an important contribution to providing insight regarding variables that are associated with chasing among older-aged gamblers, one of the least represented populations of gamblers in the literature. The results suggest that specific training on mentalizing abilities could help gamblers to reflect on their own behaviors in terms of mental states, rather than following the impulse to gamble in order to ameliorate poor mood.

## 1 Introduction

Epidemiological studies have shown that between 0.1% and 5.8% of the adult general population worldwide suffer from problematic gambling (1). Among these, an increasing proportion are older aged individuals (2). In fact, gambling participation rates among the elderly have risen, to the point that older-aged people have been identified as an at-risk group of developing gambling-related problems (2), due to several interacting risk factors that can contribute to causing gambling problems among this age cohort. From an environmental perspective, a lack of familiar and/or social support (3), stressful life events (e.g., widowhood and retirement) (4), and poor social adjustment (5) can have a significant impact on gambling participation. From a psychological perspective, high levels of impulsivity, deficits in coping strategies and problem-solving, and negative affectivity (e.g., worry, anxiety, and stress) could lead to problematic gambling, as a way to soothe emotional suffering (6). Moreover, neurobiological modifications, mainly involving the frontal areas of the brain, may result in deficits in executive functions, leading to impairments in inhibition response, planning, and decision-making (7; see also 8, 9).

Among the most characterizing features of disordered gambling is chasing behavior. It consists of the drive to invest increasing amounts of money in gambling after losses in an attempt to recoup the money that was previously lost. In other words, instead of serving as a deterrent, gambling losses represent an incentive for continued gambling, and has been recognized as a behavior that facilitates problematic gambling involvement (10, 11). For some scholars, chasing has been conceptualized as a behavioral equivalent to substance seeking (12) and represents a pathognomonic feature of disordered gambling which occurs among approximately 80% of individuals with a problematic gambling involvement (13) and is a key risk factor in the transition from recreational to disordered gambling (14–17). Chasing losses represents a directly observable and measurable diagnostic criterion of gambling disorder, and is an idiosyncratic characteristic in the field of addictions, that differentiates gambling disorder from substance use disorders (18, 19).

Criterion 6 for gambling disorder in the DSM-5 (14, 20) refers to chasing (“After losing money gambling, often returns another day to get even [“chasing” one’s losses]”). This refers exclusively to chasing *losses*, and to *between-session* chasing, namely to return another day to recoup gambling losses. However, recent studies have broadened this construct, showing how chasing concerns not only losses but also wins, with the hope of earning more (21–23), and that chasing behavior is realized not only by returning another day to gambling but also occurring in the same gambling session while it is still ongoing, i.e., *within-session* chasing (24; see 25 for a review). Chasing plays a prominent role in several theoretical models, such as the pathways model (26), according to which, chasing of both wins and losses, is recognized as a consequence of classical and operant conditioning, as well as a factor that, across the different pathways, leads to gambling disorder. In other words, engaging in chasing can triple the risk of developing disordered gambling (27).

The importance of chasing in maintaining gambling involvement is evidenced by the number of studies over the past decade that have focused upon the investigation of the endophenotypes, both gambling- and personality-related, that contribute to chasing behavior. The majority of research studies that have empirically investigated chasing behavior have highlighted the role of different personality features mostly related to impulsivity (e.g., 24), including sensation seeking (28), disinhibition (29), and a present-oriented time perspective (30, 31).

As well as impulsiveness, gamblers exposing themselves to the risk of further losses by persisting in gambling, may be related to negative emotions. In support of this, O’Connor and Dickerson (32) conducted an interview-study involving 18 regular gamblers to investigate the factors influencing the decisions to chase within-session. They reported that chasing allowed gamblers to modulate the frustration and anger after gambling losses and to experience feelings of relief and excitement. In another study, gamblers with high levels of depression reported a significantly greater number of games played and longer duration of gambling (33). Devos et al. (34) observed an increased persistence in gambling on a simulated slot machine task after experimentally inducing sadness in a group of recreational gamblers. Although the association between negative emotions and gambling is well established, the relationship between negative emotions and chasing has not been widely explored, especially among older-aged gamblers, considering that the aforementioned studies only recruited early- or early-middle aged adults (e.g., 32–34).

Mentalizing is an ability that develops within early attachment relationships that “can protect individuals from stress-affected emotional arousal” (35, p.5), allowing an adaptive regulation of emotions (e.g., 36–38), and preventing the impulsive acting out of negative emotions with the consequent risk of engaging in problematic behaviors. Mentalizing is a concept derived from different disciplines from psychoanalysis to social cognition (38) and comprises the imaginative ability of individuals to perceive and interpret their own and others’ behaviors in terms of mental states, such as thoughts, beliefs, wishes, and desires (39–41). Mentalizing is a fundamental developmental achievement by which behavior becomes meaningful and predictable, with both interpersonal and intrapsychic implications (38, 42). On an interpersonal level, mentalizing facilitates social relationships, while on an intrapsychic level, it is associated with the development of second-order representations that allow the modification of mental states (41, 43). Consequently, mentalizing represents an important skill not only in increasing awareness of the mental states but also in constituting a fundamental requirement for the modification of the mental states themselves (40, 41). Several studies have demonstrated an association between gambling and mentalizing deficits (e.g., 44, 45) that, according to Fonagy and Bateman (46), can decline in two different forms: *hypomentalization* and *hypermentalization*. Hypomentalization consists of a difficulty in interpreting human behaviors in terms of internal mental states, while hypermentalization consists of excessive and inaccurate mentalizing. It has been demonstrated that both the deficit dimensions of mentalization are involved in gambling (47–49). These mentalizing impairments could be responsible for the confidence that gamblers have in their own performance in both gambling and non-gambling tasks, as well as for the tendency to manage emotional distress in a dysfunctional and impulsive way (45, 50, 51).

Although the association of gambling with mentalizing deficits has been previously documented among both adults and adolescents (e.g., 44, 47–49, 52, 53), to date, the role of mentalizing deficits in chasing behavior among older-aged gamblers is unknown. This aspect should instead be thoroughly investigated, especially in light of the results of some studies having observed a decline in mentalization abilities over time and, more specifically, from the age of 50 years onwards (e.g., 54, 55).

In the light of the aforementioned literature gaps, the present study empirically investigated chasing behavior among older-aged gamblers, in order to identify its psychological determinants. More specifically, the present study investigated the (previously) unexplored relationship between chasing, gambling severity, psychological distress, and mentalizing among older-aged gamblers. Based on previous chasing research, it was hypothesized that depression, mentalizing deficits and gambling severity would predict chasing behavior among older-aged gamblers.

## 2 Materials and methods

### 2.1 Participants and procedure

The sample comprised 116 gamblers (59% males), aged between 56 and 84 years (*M_age_* = 67.59 years; *SD* = 6.04). They were recruited from several Italian gambling venues, and of those approached to do so, 26% declined the invitation. Participants performed the ChasIT, a computerized task, to assess chasing behavior (56) and completed the Italian versions of the South Oaks Gambling Screen (SOGS; 57, 58) to assess problem gambling severity, the Depression Anxiety Stress Scale (DASS-21; 59, 60) to assess psychological distress, and the Reflective Functioning Questionnaire (RFQ-8; 61, 62), to assess mentalization, respectively. Half of the sample carried out the ChasIT before the paper-and-pencil psychometric scales, in order to balance any potential influence of each measure on the others. Because the ChasIT task includes three experimental conditions (control, loss, and win), each participant was randomly assigned to each condition, resulting in approximately the same number of participants being assigned to each condition (Control, N = 39; Loss, N = 40; Win, N = 38). The self-report measures were administered in counterbalanced order and, for each psychometric instrument, participants received written instructions.

The completion of both computerized task and self-report measures took place in a quiet room of the gambling venues, where each participant was individually and anonymously tested, after being informed about the general purpose of the study and having provided written informed consent. They were also assured that they could withdraw from the study whenever they wanted.

Participation in the study took approximately 25 minutes. After data collection, participants were debriefed about the real aims of the study, obtaining more detailed information about the specific hypotheses of the study. Participation in the study was voluntary and participants did not receive any reward. The present study was conducted adhering to the Helsinki Declaration and was approved by the Institutional Ethical Review Board of the first author’s university.

### 2.2 Measures

#### 2.2.1 Problem gambling

The SOGS is a self-report scale assessing gambling frequency and severity. The first section of the SOGS comprises non-scored items providing information about the frequency of participation in gambling, the largest amount of money gambled on any one day, and the preferred gambling activities (e.g., cards, horses, bingo, etc.). The second section comprises 20 scored dichotomous (*yes/no*) questions assessing the severity of gambling involvement through items that investigate the chasing behavior, the guilt related to gambling, the loss of control over gambling, etc. The scores range from 0 to 20, with higher scores reflecting more severe gambling involvement. More specifically, scores from 0 to 2 indicate no gambling problems, scores of 3 and 4 indicate problem gambling, and a score of 5 or above denotes (probable) pathological gambling. In the present study, the SOGS had very good internal consistency (α = 0.83).

#### 2.2.2 Psychological distress

The DASS-21 is a 21-item self-report measure assessing three negative psychological mood states (i.e., depression, anxiety, and stress) during the past two weeks. Items rated on a four-point Likert scale ranging from 0 (*did not apply to me at all*) to 3 (*applied to me very much, or most of the time*). The scores range from 0 to 63 for the whole scale, and 0 to 21 for the three subscales. Higher scores reflect greater psychological distress. In the present study, the full DASS-21 had excellent internal consistency (α = 0.93) and the subscales had very good internal consistency: depression (α = 0.83), anxiety (α = 0.84), and stress (α = 0.85).

#### 2.2.3 Mentalizing

The RFQ-8 is an eight-item self-report measure assessing two different dimensions of mentalization (i.e., certainty about mental states and uncertainty about mental states). Items are rated on a seven-point Likert scale, from 1 (*strongly disagree*) to 7 (*strongly agree*). Scores range from 0 to 28 on the two subscales (but there is no overall scale score). Low scores on the certainty subscale scale indicate inaccurate mentalizing (i.e., hypermentalizing) while high scores on uncertainty subscale reflect a lack of knowledge about mental states (i.e., hypomentalizing). In the present study, the certainty (α = 0.75) and uncertainty (α = 0.72) subscales both had very good internal consistency.

#### 2.2.4 Chasing behavior

The ChasIT is a computerized task developed with *SuperLab 4.0* experimental software that assesses chasing behavior frequency. It simulates a card game in which participants play against the house with a virtual amount of money (€10) that participants are asked to treat as real money. Each of the 60 trials consists of the presentation of the back of two cards, one from the player and one from the house. Each card reports a number ranging from 1 to 9. For each trial, if participants have the highest card they win €1, whereas if they have the lowest card they lose €1. Unbeknownst to the participants, gambling outcomes were predetermined whereby the rate of winning and losing trials depend upon the experimental conditions: in the control condition, after the first half of the task, participants keep the entire budget; in the loss condition, participants lose more than the initial budget (i.e., €12); in the win condition, participants win more than the initial budget (i.e., €12). However, in all three conditions, for each of the subsequent 30 trials, participants are allowed to continue or to stop gambling and were informed about the amount of money remaining. Participants who choose to stop gambling at the beginning of the second phase of the task are classified as “non-chasers”, whereas participants who decide to continue gambling are classified as “chasers”. Both the decision to chase and the number of trials played are measures of chasing behavior.

### 2.3 Statistical analysis

Data were analyzed with the *IBM Statistical Package for the Social Sciences, version 20.0*. The alpha significance level was set at *p*<.05. All variables were initially screened for missing data, distribution abnormalities, and outliers (63). Because the distributions of chasing frequency and SOGS were positively skewed, square-root transformation was performed on these variables, so that the assumptions of normality, linearity, and homoscedasticity were adequately met. Correlational analyses were performed to examine the relationships between the study variables. Chi-square tests were used to assess differences in percentages for categorical data. Analysis of variance was used to assess mean differences on continuous variables. Logistic and linear regression analyses were performed to identify the predictors of chasing behavior. To control for the presence of multicollinearity, before interpreting the regression coefficients, the variance inflation factor (VIF) was calculated. In the present study, the VIF was below the recommended cut-off of 2.5 (64), indicating no issues with multicollinearity.

## 3 Results

Most of the sample only gambled offline (77%), whereas the 7.8% preferred online gambling, and 14.7% participated in both online and offline gambling. More than one-third of the sample reported gambling onset before the age of 18 years (38.8%), and the 70.7% before the age of 30 years, with only 5.3% having started gambling at the age of 60 years or over. The most reported gambling types (participants could report more than one type of gambling) were buying lottery tickets (95.8%), gambling on card games (46.3%), and sports betting (40.6%). The most popular places to gamble were tobacco shops (47%), home (21.9%), bars (26%), and betting centers (12.5%). Participants preferred gambling with friends (49%) or alone (21.9%). The most reported motivations for gambling (participants could report more than one motivation) were: entertainment (31.3%), money (20.8%), socializing (13.5%), and hobby (10.4%). **Table 1** reports the socio-demographic variables of the overall sample.

**Table 1:** Socio-demographic variables of the total sample.

	Range	Total sample (N = 116)	
		M (*SD*)	
**Education**	5-18	11.21 (4.06)	
		*N*	%
**Professional status**	Employed	41	35.3
	Unemployed	21	18.1
	Retired	54	46.6
**Education**	Primary School diploma	16	13.8
	Middle School diploma	33	28.4
	High School diploma	50	43.1
	Master’s degree	17	14.7
**Marital status**	Single	6	6.4
	Live-in partner	2	2.1
	Married	63	67
	Separated	9	9.6
	Widower	14	14.9

Correlational analysis showed that both SOGS and chasing behavior (ChasIT performance) (i) positively correlated with all three DASS-21 subscales, and the uncertainty subscale of the RFQ-8, and (ii) negatively correlated with the certainty subscale of the RFQ-8. SOGS scores and chasing behavior were positively correlated each other (see **Table 2**).

**Table 2:** Pearson correlation coefficients among measures.

	2	3	4	5	6	7
1. SOGS	.401**	.199*	.273**	.300**	-.287**	.365**
2. Chasing	-	.364**	.279**	.375**	-.375**	.296**
3. DASS-21 Depression		-	.664**	.678**	-.439**	.385**
4. DASS-21 Anxiety			-	.652**	-.308**	.360**
5. DASS-21 Stress				-	-.355**	.315**
6. RFQ-8 Certainty					-	-.566**
7. RFQ-8 Uncertainty						–

* Correlation is significant at the *p*< 0.05 level (2-tailed); ** Correlation is significant at the *p*< 0.01 level (2-tailed)

SOGS, South Oaks Gambling Screen; DASS-21, Depression Anxiety Stress Scale; RFQ-8, Reflective Functioning Questionnaire.

To verify the presence of any differences between participants randomly assigned to the different experimental conditions of the ChasIt task on the examined variables (gender, age, education, SOGS, DASS-21, and RFQ-8 scores), the data were analyzed using either χ^2^ test or univariate ANOVA. Mixed ANOVA was performed on RFQ-8, given that the scale does not have an overall total score. The results indicated that the three groups did not differ in terms of gender (χ^2^ [2]= 4.54; *p*= 0.10) and age (*F*_2,113_ = 0.21; *p*= 0.81), as well as scores on the SOGS (*F*_2,113_ = 1.64; *p*= 0.20), DASS-21 (*F*_2,113_ = 0.54; *p*= 0.58), and RFQ-8 (*F*_2,113_ = 0.14; *p*= 0.87), except for education (in years, *F*_2,113_ = 3.25; *p*<.05), with participants in the control group reporting a significantly lower number of years of education.

Similarly, to ascertain whether SOGS, DASS-21, and RFQ-8 scores, and ChasIT performance varied by gender, analysis was carried out using χ^2^ test or univariate ANOVA. Mixed ANOVA was performed on RFQ-8, given that the scale does not have an overall total score. The results indicated no significant gender differences for scores on the DASS-21 (*F*_1,114_ = 0.06; *p*=0.80), and RFQ-8 scores (*F*_1,114_ = 0.13; *p*= 0.72), as well as on the decision to chase (χ^2^ [1]= 1.62; *p*= 0.20) and chasing frequency (*F*_1,114_ = 0.52; *p*= 0.47). However, there was a significant gender difference in SOGS scores (*F*_1,114_ = 11.01; *p*<.01, η²*_p_* = 0.09), with males reporting higher scores than females. The descriptive statistics by gender and ChasIT experimental conditions are shown in the **Table 3**. For ease of interpretation, descriptive statistics are reported for the untransformed variables.

**Table 3:** Means and standard deviations by ChasIT experimental conditions and gender.

*Condition*	Control *(N = 39)*				Loss *(N = 39)*				Win *(N = 38)*			
*Gender*	Males *(N= 19)*		Females *(N=20)*		Males *(N= 28)*		Females (*N= 11*)		Males *(N= 21)*		Females *(N= 17)*	
	*Mean*	*SD*	*Mean*	*SD*	*Mean*	*SD*	*Mean*	*SD*	*Mean*	*SD*	*Mean*	*SD*
SOGS	1.58	2.71	0.60	1.14	2.32	2.75	1.09	1.97	2.67	3.40	0.76	1.78
DASS-21 Depression	3.42	4.92	4.65	3.76	4.75	4.56	6.44	5.57	4.86	4.73	4.53	5.27
DASS-21 Anxiety	4.26	5.39	4.30	3.18	5.07	4.77	5.44	6.29	3.68	4.01	4.69	4.28
DASS-21 Stress	4.95	4.96	6.15	4.49	6.20	5.13	6.97	6.20	7.19	5.54	4.77	3.62
RFQ-8 Certainty	1.55	0.84	1.56	0.75	1.33	0.93	1.20	0.85	1.52	0.98	1.44	0.84
RFQ-8 Uncertainty	0.36	0.40	0.39	0.35	0.61	0.54	0.76	0.61	0.58	0.47	0.46	0.56

SOGS, South Oaks Gambling Screen; DASS-21, Depression Anxiety Stress Scale; RFQ-8, Reflective Functioning Questionnaire.

To verify whether SOGS, DASS-21, and RFQ-8 scores, and ChasIT performance varied by age of onset of gambling involvement, analysis was carried out using χ^2^ test or univariate ANOVA using the four groups of gambling onset (before 18 years, between 18 and 30 years, between 31 and 59 years, and 60 years or over) as the grouping variable. Mixed ANOVA was performed on RFQ-8, given that the scale does not have an overall total score. The results indicated no significant differences for scores on the SOGS (*F*_3,112_ = 2.05; *p*= 0.11), DASS-21 (*F*_3,112_ = 0.76; *p*= 0.52ns), and RFQ-8 scores (*F*_3,112_ = 1.43; *p*= 0.24), as well as on the decision to chase (χ^2^ [3]= 2.69; *p*= 0.44) and chasing frequency (*F*_3,112_ = 0.13; *p*= 0.94).

To ascertain whether the decision to chase after the first phase of the ChasIT task was affected by the experimental condition, χ^2^ was conducted. The analysis showed that the decision to continue gambling did not vary as a function of experimental condition (χ^2^[2] = 4.10; *p*= 0.13). Furthermore, to verify if chasing frequency (i.e., the number of trials played during the second phase of the ChasIT task) was affected by the experimental condition, univariate ANOVA was conducted. The analysis showed that the chasing frequency did not vary as a function of experimental condition (*F*_2,113_ = 1.31; *p*= 0.27).

Of the total sample, 29.3% decided to continue gambling in the second half of the ChasIT task for an average number of 12.73 trials played (*SD*= 10.80). Based on the decision to chase, participants were divided into two groups: chasers and non-chasers. Analyses showed that the two groups did not differ in gender (χ^2^ [1]= 1.62; *p*= 0.20) or age (*F*_1,114_ = 0.56; *p*= 0.45), but did on SOGS scores (*F*_1,114_ = 15.88; *p*<.001; η²*_p_* = 0.10). Chasers reported higher problem gambling scores on the SOGS than non-chasers. All subsequent analyses were therefore performed controlling for gambling severity.

Chasers and non-chasers were also compared on negative affectivity and mentalizing scores. The ANCOVA performed on the DASS-21 subscales, using decision to chase as the group variable (controlling for SOGS scores) showed no effect for negative affectivity (*F*_2,112_ = 3.06; *p*<.05) but a significant main effect for SOGS scores (*F*_1,113_ = 6.71; *p*=.01; η²*_p_* = 0.05), and significant interaction effect for negative affectivity with chasing group (*F*_2,112_ = 5.58; *p*<.01; η²*_p_* = 0.09). No main effect for the chasing group (*F*_1,113_ = 2.94; *p*= 0.09) and no interaction for negative affectivity with SOGS scores (*F*_2,112_ = 2.29; *p*= 0.11) were found. The results indicated greater level of depression and stress among chasers, as compared to non-chasers. The difference remained significant even after controlling for gambling severity.

An ANCOVA was also performed on the RFQ-8 subscales, using the decision to chase as the group variable (controlling for SOGS scores). This showed a significant main effect for mentalizing (*F*_1,113_ = 31.99; *p*<.001; η²*_p_* = 0.22), and significant interactions for mentalizing with both SOGS (*F*_1,113_ = 11.12; *p*=.001; η²*_p_* = 0.09) and chasing group (*F*_1,113_ = 6.47; *p*=.01; η²*_p_* = 0.05). No significant main effects for both SOGS (*F*_1,113_ = 0.70; *p*= 0.40) and chasing group (*F_1,113_ =* 2.79; *p*= 0.10) were found. The results indicated greater levels of mentalizing deficits (in the direction of hypermentalizing) among chasers, as compared to non-chasers. The difference remained significant even after controlling for gambling severity.

To evaluate the contributions of gender, age, education, chasing task condition, SOGS scores, DASS-21 and RFQ-8 subscales to chasing behavior, a hierarchical logistic regression analysis was conducted, using decision to chase as the criterion variable. The results of the final regression model indicated that depression and anxiety subscale of DASS-21 and SOGS scores significantly predicted chasing decision (χ^2^[3, *N*= 116] = 24.19; *p*<.001). The overall model explained 27% of variance (Nagelkerke R^2^). The overall classification accuracy was 75.9% (see **Table 4**).

**Table 4:** Results of hierarchical logistic regression analysis on the decision to chase.

Predictors	*B*	*SE*	Wald statistic	*df*	*p* value
*Step 1*					
**SOGS**	1.05	0.33	9.88	1	0.002
*Step 2*					
**SOGS**	1.23	0.36	11.90	1	0.001
**DASS-21 Depression**	0.20	0.07	9.33	1	0.002
**DASS-21 Anxiety**	-0.14	0.07	4.09	1	0.043

SOGS, South Oaks Gambling Screen; RFQ-8, Reflective Functioning Questionnaire.

A hierarchical linear regression analysis was also carried out on ChasIT total score (chasing frequency), with gender, age, education, chasing task condition, SOGS scores, DASS-21 and RFQ-8 subscales as independent variables. SOGS, depression subscale of DASS-21 and certainty subscale of RFQ-8 emerged as significant predictors of chasing frequency, with the overall model explaining nearly 30% of the total variance (R^2^ *^adj^* = 0.28; *F*_3,112_ = 15.91; *p* <.001) (**Table 5**).

**Table 5:** Results of hierarchical linear regression analysis on the chasing persistence.

Predictors	B	*SE*	β	*t*	*p* value	VIF
*Step 1*						
**SOGS**	0.90	0.17	0.44	5.20	0.000	1.00
*Step 2*						
**SOGS**	0.78	0.17	0.38	4.62	0.000	1.04
**DASS-21 Depression**	0.08	0.02	0.28	3.45	0.001	1.04
*Step 3*						
**SOGS**	0.68	0.17	0.33	3.94	0.000	1.12
**DASS-21 Depression**	0.06	0.02	0.21	2.33	0.021	1.25
**RFQ-8 Certainty**	-0.31	0.14	-.20	-2.18	0.031	1.33

B, unstandardized coefficient; β, standardized regression coefficient; VIF, Variance Inflation Factor; SOGS, South Oaks Gambling Screen; DASS-21, Depression Anxiety Stress Scale; RFQ-8, Reflective Functioning Questionnaire.

## 4 Discussion

The aim of the present study was to investigate chasing behavior among older-aged gamblers, using an experimental task that is frequently adopted in the literature and has demonstrated good construct validity (29–31, 53, 56, 65, 66), allowing the overcoming of all the limitations of self-report measures (67). More specifically, the study focused on the previously unexplored relationship between chasing, gambling severity, negative affectivity, and mentalizing among older-aged gamblers.

Firstly, the results of the present study showed that the ChasIT conditions (i.e., win, control, and loss, to which each participant was randomly assigned) had no effect on chasing behavior, neither on the decision to continue betting, nor on the frequency of trials played in the task. In other words, participants decided to continue or not continue gambling and how many trials to play irrespective of previous gambling outcomes. While this result may appear odd in light of the fact that DSM-5 diagnostic criteria for gambling disorder explicitly refers to the effect of losses on chasing behavior (14), this observation was not unexpected, given that the majority of studies investigating the role of gambling outcomes (both win and losses) on subsequent decision to persist in gambling have reported no effect of loss or win conditions (30, 31, 56, 65; for a review, see 25). This result, which is echoed in most previous studies, can be interpreted as evidence in support of what was previously suggested by Nigro et al. (29), where chasing behavior represents a personality trait-like characteristic, which therefore disregards contextual variables such as previous gambling outcomes, but is only affected by variables intrinsic to the gambler’s personality, such as impulsivity-related characteristics.

In support of this, past studies have demonstrated strong associations of chasing with both delay discounting (65), foreshortened time horizon (30), alcohol consumption (53), cognitive distortions (66), and subjective feeling of craving (31). In summary, the lack of effect of previous gambling outcomes on chasing behavior could indicate that endophenotypic characteristics have greater weight in incentivizing gamblers to chasing behavior as compared to contextual variables (such as previous wins or losses). However, this result was in contrast to a wealth of studies that, interviewing people about their loss-chasing behavior, showed a frequent continuing and/or intensifying gambling in the face of losses (e.g., 17, 27, 68–72). Regarding this, it cannot be ruled out that within-session chasing (assessed using the ChasIT), i.e., the persistence in the same gambling session, and loss-chasing between-session, i.e., returning another day to gamble, although strongly related, are not overlapping constructs. Moreover, results obtained from the laboratory studies are somewhat inconsistent. Although the majority of studies failed to support this effect (22, 30, 31, 56, 65, 66, 73), a handful of studies have shown that gamblers play more extra trials after losses (29, 74, 75) or wins (51). As suggested by a recent review (25), laboratory study results may mainly depend on the operationalization of the ‘chasing’ construct, on the characteristics of the task used, as well as on the level of gambling severity of the sample recruited.

It should be noted that chasing varied as a function of problem gambling severity. More specifically, participants who decided to chase reported more severe gambling involvement. These results corroborate what was previously found in literature by Yakovenko (75) that, assessing chasing persistence in a laboratory-based slot machine task, found higher levels of persistence among those whose gambling was disordered, compared to those whose gambling was social. Similarly, Auer and Griffiths (76), in a behavioral tracking study involving online casino players, found that chasing varied as a function of problem gambling severity. These findings are further confirmed in regression analyses where severe gambling involvement was found to be a predictor of both the decision to chase and chasing frequency. It is difficult to determine the directionality of this association: individuals may start chasing when their gambling involvement become problematic or could they may develop problem gambling because of repeated attempts to recover losses. Either way, the present study’s results confirm the importance of chasing as an indicator of a severe gambling involvement.

Interestingly, chasers reported higher levels of depression and stress than non-chasers, indicating that chasing, as well as being an indicator of more severe gambling involvement, could be a risk factor for other mental health disorders and/or symptoms. The co-occurrence of gambling disorder with other mental health disorders, including mood disorders (e.g., 9, 77), is very common. Some scholars argue that gambling disorder is a dual disorder due to the difficulty of recognizing gambling as a single nosological entity (78–81).

In general, the present pattern of results (no effect of previous gambling outcomes but an effect of gambling severity on chasing behavior) strongly resemble those of Lister et al. (73). Using a different task (i.e., a slot machine in an immersive virtual casino where gamblers played in a loss or in a win condition), they did not find a significant impact of previous outcomes on chasing persistence and decision to chase but found that participants with problem gambling and those motivated to win money were more likely to chase and gambled for more trials.

With regard to psychological distress, the present study is the first to show that negative affectivity is among the factors that predict chasing behavior. High levels of anxiety and depression were associated with the decision to chase, with depression also being associated with a larger number of trials played. Studies investigating gambling-related motivations have observed that one of the most reported is the relief from negative psychological states (21, 82, 83). Given the frequent association of gambling with negative affectivity (e.g., 49, 84–89; see 90 for a review), alexithymia (e.g., 91, 92), and emotional dysregulation (e.g., 44, 93), gambling may serve not only to ameliorate mood states (e.g., 21, 94) but also to experience excitement, and relieving boredom (95). In fact, individuals who are emotionally dysregulated could react to negative emotions engaging in impulsive behavior, such as persevering in gambling participation (96; see 97 and 98 for reviews), consequently using gambling as a kind of dysfunctional coping strategy. Indirect confirmation of this association also comes from the wealth of studies that have indicated coping as the most reported motivation to gamble, as well as a risk factor for the development of problematic gambling (21, 99–102). The negative reinforcement resulting from mood amelioration provides even more incentive for gambling participation (103) and raises the risk of disordered gambling (104, 105). In the specific field of chasing research, the present findings resonate with those that have identified a significant positive correlation between alexithymia (i.e., the difficulty in processing emotional information) and within-session loss chasing. More specifically, in two laboratory-based studies, Bibby (106) and Bibby and Ross (107) found increasing stake sizes after losses among participants with high levels of alexithymia.

Interestingly, as the regression analyses showed, mentalizing deficits contributed to chasing frequency, complementing a previous study investigating mentalizing ability among those with gambling problems (53). The present study also highlighted that hypermentalizing is an important predictor of chasing frequency among older-aged gamblers. The fact that hypermentalizing (which refers to excessive but inaccurate mentalizing) was associated with a greater number of trials played resembles the same phenomenon that has been observed in previous studies (50, 51), where participants with gambling problems, while performing worse than controls by making disadvantageous decisions, were so confident in their performance to the point that they were willing to bet on the quality of their own decisions. This lack of self-awareness in both gambling and non-gambling situations of decision-making, combined with an individual’s overconfidence in their own abilities, could push the gambler to persist in gambling in the belief that they will be able to get even. Moreover, the present study’s results further corroborate the importance of mentalizing in maintaining gambling problems (44, 47–49, 52, 53), and also extending its role as a risk factor for problem gambling among older-age gamblers.

Taken together, the present results indicate that depression and anxiety may motivate the decision to continue gambling irrespective of previous gambling outcomes in the attempt to ameliorate poor mood. The inability of gamblers to mentalize and, therefore, to reflect on their behavior and understand their underlying mental states, may explain the increased frequency of chasing, (i.e., why gamblers are unable to stop this dysfunctional behavior that, over time, is strongly associated with a problematic gambling involvement).

As for the study’s limitations, a non-clinical sample was used, although it must be considered that almost all the gamblers in the sample were familiar with playing cards, in line with the task that was used (ChasIT). Furthermore, the low sample size and potential selection bias in the recruitment of the sample are also potential limitations. It should also be noted that some of the psychometric scales used have not been specifically validated among older population. Moreover, the card game adopted to assess chasing behavior had limitations regarding the present study’s ecological validity, for different reasons. First, the small bets used may have been unattractive, given winning money is among the most reported motivations to both gamble and chasing (73; see also 108). Second, even if the influence of the type of rewards in influencing behaviors in the gambling task was unclear (e.g., 109–112), it should be noted that ChasIT task used non-real money. All these structural characteristics of the task might ultimately reduce the generalizability of the present findings. Finally, it should be noted that (i) a large proportion of gamblers now play online, whereas in the present study only a small proportion of the sample preferred gambling online; and (ii) elderly individuals constitute a heterogeneous group regarding gambling phenotypes (113), therefore, the present findings cannot be not generalized to all old-age gamblers, especially in the light of the observation that, in the present study, only 5.3% started gambling at the age of 60 years or older. It appears rare that individuals start gambling in old age. In general, given the small effect sizes, the present results should be interpreted cautiously, suggesting that the relationship between the study variables should be investigated using larger samples. Moreover, future studies should also compare the endophenotypic characteristics of older gamblers who start gambling at an older age with those who start at a young age.

The present study is the first to assess the role of gambling severity, psychological distress, and mentalizing in chasing behavior among older-age gamblers. The results demonstrated that depression and hypermentalizing, along with high levels of gambling involvement, contributed significantly to chasing behavior among the older age cohort, which, in turn, maintain problem gambling. In the light of gambling literature, it is conceivable that, in conditions of a compromised mentalization, depressed gamblers seek relief by persevering in gambling. Considering the close relationship between the ability to mentalize and the regulation of the emotional states, with mentalizing predicting both adaptive and maladaptive emotion regulation strategies (35), the present study’s results suggest that a specific training on mentalizing abilities is needed. This could help gamblers to acquire more awareness of their internal states and to reflect on their own behaviors in terms of mental states, through a process of reflection rather than following the impulse to gamble. Individuals reflecting on their own behaviors in terms of mental states involves recognizing the motivations underlying gambling behavior, as well as the choice to chase losses. Given the study’s findings, the ability to mentalize allows the opportunity to break the vicious cycle that might lead gamblers to gamble when they are depressed, finding alternative and more functional ways to improve their mood. Such interventions could have the potential to offset the trajectory toward the disorder among old age gamblers (as well as gamblers of any age).

Despite the limitations, the findings of the present study make an important contribution, providing insight into the variables that, among older-aged gamblers, contribute to chasing that is a key risk factor in several theoretical models of gambling disorder, as well as a crucial transition point from initial gambling involvement to the development of gambling disorder.

## Data Availability

The raw data supporting the conclusions of this article will be made available by the corresponding author upon reasonable request.
